# Vasopressin in the pediatric cardiac intensive care unit: Myth or reality

**DOI:** 10.4103/0974-2069.52814

**Published:** 2009

**Authors:** Vishal K Singh, Rajesh Sharma, Amit Agrawal, Amit Varma

**Affiliations:** Escorts Heart Institute and Research Center, Okhla Road, New Delhi, India; 1 Fortis & Escorts Heart Institute and Research Center Limited, Okhla Road, New Delhi, India

**Keywords:** Low cardiac output, therapy, vasopressin

## Abstract

Pediatric cardiac surgery is undergoing a metamorphosis, with more and more critical patients being operated in our country today. Although the principles of physiology have not changed, it is imperative that care providers continue to stay abreast with developments and newer drugs that may help modify the outcome. The team dynamics have also become more complex, which necessitates the need for all care providers (surgeons, cardiologists, anesthesiologists, and intensivists) to better understand the interactions and benefits of newer drugs. Vasopressin has been used in our adult patients for more than a decade and recently has found its rightful place in the pediatric armoury. The objective of this article is to review the physiology of vasopressin and the rationale of its use in critically ill children with shock, in context of the available published data.

## INTRODUCTION

Shock and hemodynamic instability are life threatening problems in pediatric cardiac critical care. Volume resuscitation based on the existing filling pressures and the underlying cardiac physiology is still the mainstay of therapy. In addition, catecholamines and other inotropic agents are essential for maintaining blood pressure and vital organ perfusion. However, catecholamines may increase the heart rate, increase myocardial oxygen consumption, compromise end organ perfusion by increasing systemic vascular resistance, and prolonged infusions of high dosage may eventually impair myocardial performance.[1] Decreased vascular and myocardial sensitivity to catacholamines has been demonstrated in shock, leading to serious adverse effects instead of the desired clinical effects.[2] Thus, there is a need for continuous evaluation of alternative and adjunctive therapies targeted at specific pathophysiological pathways to reverse shock.

Vasopressin has been used for treatment of diabetes insipidus and gastrointestinal bleeding. In the past decade, use of low-dose vasopressin as a potent vasopressor has aroused renewed interest. This has primarily emerged from its successful use in patients with cardiac arrest and in patients with vasodilatory shock.

The objective of this article is to review the physiology of vasopressin and the rationale of its use in shock and critically ill children in the context of available data that is published.

## PHYSIOLOGY OF VASOPRESSIN

Vasopressin is synthesized in the hypothalamus as prohormone preprovasopressin. Preprovasopressin is degraded to pro-vasopressin, which migrates along the neuronal axons to the posterior pituitary and is subsequently released in three fragments: vasopressin, neurophysin-II, and copeptin. Most of the newly synthesized vasopressin is stored intracellularly, and only 10 to 20 percent of the total hormonal pool within the posterior pituitary can be readily released under appropriate stimuli.[3] Once secreted in the circulation, vasopressin is accompanied by its carrier protein, neurophysin-II, which does not have any independent biological activity. Vasopressin is cleaved by vasopressinase with a half life of approximately 15 minutes, and hence it is administered by continuous infusion for the management of vasodilatory shock.[4] Vasopressin has multiple physiological functions with the most pronounced being constriction of vascular smooth muscle and osmoregulation. Vasopressin has many other physiological functions including effects on memory, sleep cycle, temperature regulation, hemostasis, insulin, and corticotrophin release.

**Figure 1:**
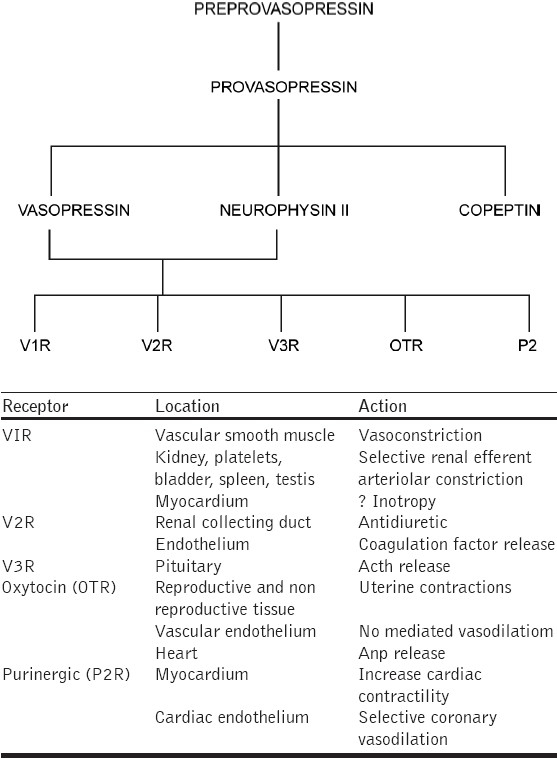
Flow diagram depicting synthesis, release and mechanism of action of arginine-vasopressin

The diversity of its actions are related to the location and density of tissue-specific G protein-coupled vasopressin receptor subtypes, which are currently classified into V1 vascular, V2 renal, V3 pituitary, oxytocin, and P2 purinergic receptors [Figure 1].[5]

Purinergic P2 receptors are located in the cardiac endothelium and may play a role in cardiac contractility and selective coronary vasodilatation. Vasopressin is important in the control of water balance and vasomotor regulation, and is also actively involved in coordinating the autonomic and endocrine responses to homeostatic disturbances

## REGULATION OF VASOPRESSIN SECRETION

The normal plasma vasopressin concentration in hemodynamically stable patients ranges between 1 to 7 pg/ml, depending on the level of hydration and osmolality. The most important stimuli for vasopressin release are increased plasma osmolality, hypotension, and hypovolemia.[6] Vasopressin release is also under the control of the sympathetic nervous system. Under resting conditions or when stretched, baroreceptors inhibit vasopressin secretion. Decreased activity due to low blood pressure decreases baroreceptor neuronal output and results in the release of vasopressin from the hypothalamus.

Other important secretagogues of vasopressin include endotoxin and proinflammatory cytokines, that is, interleukin 1β (IL-1β), interleukin-6, tumor necrosis factor-α (TNF-α).[7,8] Other nonosmotic stimuli-like pain, nausea, hypoxia, anesthetic agents, and various endogenous and exogenous chemicals, such as, norepinephrine and acetylcholine, may also trigger vasopressin release.[9,10]

A wide variation has been observed in vasopressin levels, primarily due to the fact that mature vasopressin is unstable, and has a short half life. Copeptin, a stable peptide of the vasopressin precursor, is secreted in an equimolar ratio, with more stable plasma levels and has been proposed as a more sensitive and potential prognostic marker in patients with sepsis and shock.[11] Muller *et al.* assessed the circulating levels of the copeptin in lower respiratory tract infections (LRTI), a precursor of sepsis, and concluded that copeptin levels increase with the increasing severity of LRTI, mainly in patients with community acquired pneumonia (CAP), with an unfavorable outcome.[11] The authors suggested that copeptin can be used as a novel marker for risk stratification in CAP. Morgenthaler *et al.,* also evaluated the plasma copeptin concentration in two independent studies; first in an experimental baboon model with hemorrhagic shock, and second in a prospective observational study of 101 consecutive critically ill patients at a university hospital.[12] In a logistic regression model, the serum copeptin level was the only independent significant predictor of the outcome. Copeptin concentrations were elevated in hemorrhagic septic shock. Copeptin was higher on admission in nonsurvivors as compared to survivors, suggesting copeptin as a prognostic marker in sepsis. However, this study by Morgenthaler *et al.* had a limitation, as mature arginine vasopressin (AVP) was not measured immediately in patients in the intensive care unit (ICU) and hence the AVP levels and copeptin levels could not be compared. Jochberger *et al.* also evaluated the course of copeptin and vasopressin in a patient with severe septic shock and reported that they were substantially increased during the initial 36 hours of shock. Subsequently both levels declined and exhibited another peak in response to extubation.[13]

## ROLE OF VASOPRESSIN IN SEPTIC SHOCK

### Rationale

Sepsis is the most common cause of vasodilatory shock. In sepsis, the infecting organism, and its toxins induce release of inflammatory mediators from the blood cells, vascular endothelium, the central nervous system, and the neuroendocrine system. Inflammatory mediators stimulate the inducible form of nitric oxide (NO). Nitric oxide activates soluble guanylate cyclase, which elevates intracellular cyclic guanosine 3'4' – monophosphate (cGMP) and leads to smooth muscle relaxation and vasodilatation.[14] Nitric oxide, through c-GMP is a powerful neurohumoral inhibitor of vasopressin.[15] Activation of K^+^-ATP channels by nitric oxide results in smooth muscle relaxation and also contributes to vasodilatation in shock. The cytokines may also contribute to vasopressin receptor downregulation and decreased sensitivity to the exogenous hormone.[15]

A biphasic vasopressin response takes place in vasodilatory septic shock wherein high levels are observed initially, in the early phase of hypotension, followed by inappropriately low levels as shock progresses. This may be explained on the basis of the depletion of neurohypophyseal stores, impaired baroreflex-mediated release of vasopressin, attributable to autonomic dysfunction, and downregulation of vasopressin production by increased central nitric oxide production.[16] Moreover, elevated levels of norepinephrine (endogenous and exogenous) have a central inhibitory effect on vasopressin release.[17]

Vasopressin binds to the vasopressin-specific membrane-bound V1 receptors (AVPR1A) in the vascular smooth muscle and lead to vasoconstriction. Binding to AVPR1A stimulates the activation of protein kinase C, through two second messengers, and leads to an increase in intracellular free calcium, resulting in vascular smooth muscle contraction. Vasopressin also increases the vascular tone in vasodilatory shock by closing K^+^ATP channels.[18] Vasopressin also restores the vascular tone in septic shock by ameliorating the increased cyclic GMP levels, by decreasing inducible nitric oxide synthase and thereby altering the nitric oxide cascade. Low-dose vasopressin infusion concurrently causes vasodilatation in pulmonary, cerebral, and coronary circulations via oxytocin receptor stimulation, and endothelial NO release. Low-dose vasopressin is hence more beneficial compared to catecholamine precursors in preserving vital organ perfusion. Paradoxically, vasopressin (antidiuretic hormone) increases urine output and creatinine clearance in patients with septic shock.[19] This is attributed to V1 receptor-mediated, selective renal efferent arteriolar constriction. Vasopressin is a potent stimulator of adrenocorticotropic hormone and cortisol release.[20] This is important, given the prevalence of adrenocortical dysfunction in the critically ill population. Vasopressin is also reported to mediate atrial natriuretic factor and angiotensin II secretion, as well as, stimulate prolactin and endothelin 1 release. The latter effect on prolactin secretion supposedly plays a significant role in the cellular immune response.[21]

### Published evidence

Landry *et al.* observed that some patients with advanced vasodilatory septic shock had inappropriately low plasma levels of vasopressin (3.1 ± 1 pg/ml).[22] Exogenous infusion of 0.01 units/min of vasopressin in two patients increased vasopressin levels to 27 pg/ml and 34 pg/ml, indicating that low vasopressin levels in patients with septic shock were due to impaired vasopressin secretion. Additionally septic shock patients are exquisitely sensitive to low-dose vasopressin.[22] Ten patients received vasopressin at 0.04 units/min, which increased the plasma concentration to 100 pg/ml, increased systolic blood pressure from 92 to 146 mm/Hg, increased systemic vascular resistance by 79% and decreased cardiac output by 12%. Reduction of vasopressin infusion in these patients to 0.01units/min resulted in plasma levels of 30 pg/ml, and discontinuation of vasopressin treatment in these patients resulted in a sudden decrease in arterial pressure, highlighting the increased sensitivity to low-dose vasopressin.

Malay *et al.* evaluated the effect of vasopressin on 10 patients admitted to the trauma ICU with vasodilatory septic shock.[23] They found that the use of vasopressin was associated with a significant increase in systolic blood pressure and resulted in the withdrawal of all other catecholamine support. Vasopressin had no effect on the heart rate, cardiac index, and/or pulmonary artery pressure in this series. This study also highlights the increased pressor sensitivity to vasopressin, in patients with vasodilatory septic shock. Meyer *et al.* reviewed the literature on the use of arginine vasopressin (AVP) and terlipressin (TP) (long-acting analog of vasopressin with a half life of six hours) as a rescue therapy in neonates, children, and adolescents, with catecholamine refractory septic shock or cardiocirculatory arrest and analyzed 17 reports involving a total of 109 patients, ranging from 23 weeks gestation to 19 years.[24] The most common indication for either drug was catecholamine refractory septic shock. The commonly reported response following AVP/TP administration was a rapid increase in systemic arterial blood pressure, an increase in urine output, and a decrease in serum lactate. In most reports, AVP and TP had a significant impact on the required dose of other inotropes, which could be tapered off. Despite the use of AVP/TP, mortality was high (52/109). Furthermore, no definite recommendation could be given regarding its use in severe cardiogenic shock, due to limited literature.

Yildizdas *et al.* also evaluated the role of terlipressin in catecholamine-resistant septic shock in children, and concluded that although terlipressin did not affect mortality, it significantly increased the mean arterial pressure, PaO_2_/FiO_2_ ratio, and reduced the total duration of ICU stay. In this study, norepinephrine was not used, due to nonavailability in Turkey.[25]

Meyer *et al.* assessed the efficacy of arginine vasopressin as a rescue therapy in catecholamine refractory septic and nonseptic shock in extremely low birth weight infants with acute renal injury.[26] Prospective assessment of arginine vasopressin therapy in three extremely low birth weight (ELBW) infants (mean birth weight 600g ± 30 g) with catecholamine refractory septic shock and acute renal injury, was compared with three ELBW infants with nonseptic shock and acute renal injury, at a university hospital. The main outcome measures were restoration of blood pressure with adequate organ perfusion and survival at discharge. It was found that in all the three ELBW infants with catecholamine resistant septic shock, the systemic arterial blood pressure increased substantially with restoration of urine output after arginine vasopressin administration (dosage 0.035 to 0.36 u/kg/hour). In the three ELBW infants with nonseptic shock, only a transient stabilization in mean arterial pressure with restoration of urine output was observed after vasopressin therapy (dosage 0.01 to 0.35 u/kg/hour). The mortality was higher in the non sepsis group as compared to the sepsis group.

Russell *et al.* in association with coinvestigators for vasopressin and septic shock trial (VASST) compared vasopressin and norepinephrine in patients with septic shock and concluded that there was no significant difference in the 28- and 90-day mortality rate between the two groups.[27] However, this study had limitations, in that, low-dose vasopressin (0.03 units/min) was used primarily in conjunction with catecholamines to evaluate vasopressin's catecholamine sparing effect in adult patients with a mean arterial blood pressure >70 mm Hg. This excluded cases with catecholamine refractory shock.

Russell *et al.* evaluated and reviewed 16 studies of vasopressin infusion in patients with septic shock.[4] The majority of studies revealed that vasopressin infusion increased blood pressure and urine output, and decreased the dose requirement of norepinephrine. The adverse effects of vasopressin are dose-related and are due to excessive vasoconstriction secondary to excessive AVPR1A, V2 and V3 receptors, and include decreased cardiac output, arrhythmias, coronary ischemia, mesenteric ischemia, skin and digital ischemia, hyponatremia, and increased bilirubin.

Lauzier *et al.* evaluated the use of vasopressin in early hyperdynamic septic shock in adults and found that vasopressin decreased the dose requirement of norepinephrine and improved organ dysfunction (evaluated by SOFA scores), as compared to norepinephrine.[28]

Nunez *et al.* evaluated the effects of terlipressin as a rescue treatment in children with catecholamine refractory hypotensive septic shock.[29] The terlipressin dose was 0.02 mg/kg every four hours. It was observed that terlipressin treatment induced a rapid and sustained improvement in the mean arterial blood pressure, which allowed reduction of the catecholamine infusion rates after one hour in 14 out of 16 patients. Of the 16 patients, three cases died due to refractory shock, two died subsequent to withdrawal of therapy, three cases died due to refractory arrhythmias, and one due to multiorgan failure. Four survivors had sequelae, major amputations in one case, minor in two cases, and one case had a minor neurological deficit.

According to the surviving sepsis guidelines for the year 2008, vasopressin, phenylephrine, and epinephrine should not be administered as the initial vasopressor in septic shock (grade 2C).[30] Vasopressin 0.03 units/min may be added to norepinephrine subsequently, with anticipation of an effect equivalent to that of norepinephrine alone, in adults. Vasopressin has been evaluated in a number of trials in septic shock, but with no clinching evidence for the same.[31] Vasopressin can be used as a rescue therapy in pediatric septic shock patients, as described in case reports, to improve the hemodynamics in a volume-optimized patient, especially in cases with extremely low systemic vascular resistance, despite the use of norepinephrine.

## RECOMMENDATION

On the basis of the data in Table 1, it can be summarized that vasopressin does have a promising role as a rescue therapy, for a short duration, in catecholamine-resistant septic shock. Vasopressin, used in the recommended dosage range, does result in the improvement of blood pressure and urine output and allows tapering of catecholamines, but adverse effects related to vasoconstriction are a concern and hence the dosage and duration of vasopressin therapy should be strictly supervised, and more trials are required to streamline these issues.

**Table 1:** Trials evaluating the role of vasopressin in septic shock

Author, reference	Year	Setting	Total no. of patients	Study design	Findings
Landry, 22	1997	Patients with vasodilatory shock	19	Prospective case series	AVP levels are low in vasodilatory shock and AVP infusion leads to improvement in ABP
Malay, 23	1999	Septic shock/trauma	10	Randomized control trial	Improvement in ABP, decrease or discontinuation of catecholamines
Meyer, 24	2008	Catecholamine refractory shock or cardiocirculatory arrest in neonates, children, and adolescents	109	Retrospective	Increase in ABP, increase in urine output, decrease in serum lactate, and reduction in inotrope dosage
Yildizdas, 25	2008	Septic shock and refractory hypotension	58	Prospective	Increases MAP, PaO_2_/FiO_2_ ratio, decrease in HR
Meyer, 26	2006	Catecholamine refractory shock and acute renal injury in ELBW infants	6	Case series	Increase in MAP and urine output, two survivors
Russell, 27	2008	VASST trial - vasopressin versus norepinephrine in septic shock	778	Multicenter, randomized double blind trial	No reduction in mortality with low-dose vasopressin as compared to norepinephrine
Russell, 4	2007	To review mechanism of action and clinical studies of vasopressin in septic shock	16 trials analyzed	Review	Increase in MAP and urine output and decrease in norepinephrine dosage
Lauzier, 28	2006	Compare AVP and NE effects on hemodynamic variables and organ dysfunction in early hyperdynamic septic shock	23	Randomized open label, controlled	Increase in ABP, creatininine clearance, and decrease in SOFA scores with AVP
Nunez, 29	2006	Terlipressin in catecholamine refractory shock	16	Prospective cohort	Increase in MAP, decrease in catecholamine infusion. Death in nine patients

AVP - Arginine vasopressin, ABP - Arterial blood pressure, MAP - Mean arterial pressure, VASST - Vasopressin and septic shock trial, ELBW - Extremely low birth weight, NE - Norepinephrine, SOFA - Sequential Organ Failure Assessment, HR - Heart Rate

## VASOPRESSIN IN POST CARDIOPULMONARY BYPASS VASODILATORY SHOCK

### Rationale

Post cardiopulmonary bypass (CPB) catecholamine-resistant vasodilatory shock is a known entity, and vasopressin levels have a similar biphasic pattern, as in septic shock.[32] Jochberger *et al.* evaluated serum vasopressin concentration in a group of critically ill patients and concluded that AVP serum concentration 24 hours after an ICU stay, including cases after cardiac surgery, was significantly increased.[33] The authors concluded that relative and absolute AVP deficiencies were infrequent entities during acute surgical critical illness, generally remaining without significant cardiovascular effects. Forrest *et al.* reported in their review that prolonged hypovolemia, sepsis, and CPB may lead to vasopressin levels that are inappropriately low for the degree of hypotension, leading to pathological vasodilatation.[6] This may be due to exhaustion of the secretory stores in the neurohypophysis and hypothalamus, after prolonged stimulation. Impaired autonomic function has been reported in septic shock following CPB, and this may reduce the baroreceptor-mediated secretion of vasopressin.[34]

### Published evidence

Argenziano *et al.* evaluated post bypass vasodilatory hypotension in a general cardiac series and found that plasma vasopressin levels were significantly lower in vasodilated patients than in cases with cardiogenic shock.[35] In a retrospective review from the same study, 26 heart transplants and 16 left ventricular assist device cases received vasopressin infusion for vascular support. It was observed that vasopressin administration produced a significant increase in mean arterial pressure and systemic vascular resistance along with a significant reduction in noradrenaline dosage, especially in severely hypotensive patients.

Rosenzweig *et al.* have reported their experience in 11 profoundly ill infants and children aged three days to 15 years, treated with vasopressin for hypotension after cardiac surgery, which was refractory to standard cardiopressors.[32] Although underlying heart disease was present, only two patients had severely depressed cardiac function as demonstrated by 2-D echocardiography, before administration of vasopressin. All patients were intubated and were receiving multiple vasopressors and inotropes including dobutamine, dopamine, milrinone, and epinephrine. Five patients received vasopressin intraoperatively immediately after cardiopulmonary bypass, five in the ICU within 12 hours of surgery, and one on postoperative day two for hypotension associated with sepsis. The dose of vasopressin was adjusted according to patient size and ranged from 0.0003 to 0.002 units/kg/minute. During the first hour of treatment with vasopressin, systolic blood pressure rose from 65 ± 14 to 87 ± 17 mmHg and epinephrine dosage was decreased in five of eight patients. Plasma vasopressin levels before treatment were available in three patients and demonstrated arginine vasopressin depletion. All nine children with vasodilatory shock survived their intensive care stay. The two patients who received arginine vasopressin in the setting of poor cardiac function died despite transient improvement in blood pressure.

Jerath *et al.* also evaluated the clinical impact of vasopressin infusion on hemodynamics and liver and renal functions in pediatric patients, as a retrospective study, in 117 patients (85 cardiac and 32 noncardiac) requiring intravenous infusion of vasopressin for longer than 60 minutes, for advanced shock.[36] The median dose was. 0001 units/kg/min for cardiac patients and. 0002 units/kg/min in noncardiac patients. The median infusion time was 24 hours in cardiac patients and 18 hours in noncardiac patients. Both cardiac and noncardiac patients showed a significant decrease in inotrope requirement, without any change in central venous saturation or lactate during infusion. Both groups had increased urea and creatinine and decreased urine output with a longer duration/higher cumulative dose of vasopressin. There was a significant increase in conjugated bilirubin level in the noncardiac group during vasopressin infusion. The platelet count was significantly lower during infusion in both groups.

Lechner *et al.* reported a retrospective study on the effects of arginine vasopressin treatment in neonates with catecholamine-resistant systemic vasodilatation after cardiopulmonary bypass.[37] They evaluated 172 neonates who underwent open heart surgery and 17 developed vasopressor-resistant hypotension and were treated with arginine vasopressin. Thirteen of these had Stage 1 palliation of a single ventricle (Norwood procedure), two underwent the Ross procedure, and two had the arterial switch operation. All patients received multiple traditional inotropes and vasopressors prior to vasopressin administration and vasopressin was administered at a dose of 0.0001 u/kg/min to a maximum of 0.0003 u/kg/min. Arginine vasopressin resulted in a significant increase in blood pressure and also the requirement for traditional vasopressors decreased significantly. No peripheral vasoconstriction or ischemic lesions were observed. Four of the thirteen patients who underwent single ventricle palliation died.

## RECOMMENDATION

It can be summarized [Table 2] that vasopressin as a short-term rescue therapy in post cardiopulmonary bypass vasodilatory shock needs to be evaluated further, as the existing data reveals an improvement in blood pressure and a significant decrease in inotrope requirement, particularly in catecholamine-resistant clinical scenarios with vasopressin infusion.

**Table 2:** Trials evaluating role of vasopressin in post cardiopulmonary bypass vasodilatory shock

Author, reference	Year	Setting	Patient number	Study design	Findings
Argenziano, 34	1997	Vasodilatory shock post IVAD implant	10	Randomized controlled trial	Increase in ABP, decrease or discontinuation of catecholamines, inappropriately low vasopressin levels
Argenziano, 35	1998	Post bypass vasodilatory shock	40	Retrospective case series	Increase in ABP, decrease or discontinuation of catecholamines, inappropriately low vasopressin levels
Rosenzweig, 32	1999	Pediatric vasodilatory shock post bypass	11	Case series	Increase in ABP, decrease or discontinuation of catecholamines, inappropriately low vasopressin levels
Jerath, 36	2008	Pediatric advanced vasodilatory shock	117	Retrospective study	Improvement in hemodynamic status, decrease urine output, reduction in platelet count
Lechner, 37	2007	Vasopressor resistant hypotension	17	Retrospective study	Increase in ABP, decrease in requirement of traditional vasopressors

AVP - Arginine vasopressin; ABP - Arterial blood pressure; MAP - Mean arterial pressure

## VASOPRESSIN DURING CARDIOPULMONARY RESUSCITATION

### Rationale

Interest in the use of vasopressin as a therapy for ventricular fibrillation was triggered by the observation that vasopressin levels were significantly higher in resuscitated rather than in nonresuscitated patients undergoing CPR for out-of-hospital cardiac arrest.[38]

Vasopressin is superior to epinephrine for increasing vital organ blood flow, in particular coronary and cerebral blood flow, when administered intravenously as well as endobronchially or via the intraosseous route.[38]

### Published evidence

In a prospective study of 40 patients with out-of-hospital ventricular fibrillation, resistant to defibrillation, a significantly larger number of patients who received 40 units of vasopressin intravenously compared to 1mg of epinephrine, were successfully resuscitated and survived for 24 hours.[39] However, for in-hospital cardiac arrest, a triple blind randomized controlled trial failed to demonstrate a survival advantage for vasopressin over epinephrine.[40] However, this was challenged by other authors, as 50 percent of the cases had pulseless electrical activity, which had a poor prognosis. Moreover, the mean time to study drug administration in the in-hospital study was about half that of the out-of-hospital study, which may mask the potential benefit from vasopressin as suggested by the animal studies of prolonged CPR.[41] Subsequently, a comparison of vasopressin and epinephrine for out-of-hospital cardiac arrest, which included 1,186 adult patients, demonstrated a significantly better outcome among patients with asystole, who had received vasopressin, although no significant difference in the outcome was demonstrated in patients with ventricular tachycardia or pulseless electrical activity.[42] Dudkiewicz *et al.* evaluated vasopressin after traumatic brain injury for maintaining cerebral perfusion pressure and reported that intracranial pressure and brain tissue oxygenation were improved at the expense of the periphery, suggesting that vasopressin does have a role in preserving cerebral perfusion pressure in critically ill patients.[43] The European Resuscitation Council recommends 40 units of vasopressin in adults as an initial vasopressor in case of shock-refractory ventricular fibrillation, as an alternative to 1 mg of epinephrine.[44] The 2005 American Heart Association guidelines recommend 40 units of vasopressin intravenous or intraosseous, to replace the first or second dose of epinephrine in cardiac arrest, in adults.[45] A retrospective case series of children with cardiac arrest suggested that vasopressin 0.4 units/kg/dose is beneficial during prolonged pediatric cardiac arrest, following the failure of conventional cardiopulmonary resuscitation.[46] A second retrospective case series of pediatric cardiac arrests, unresponsive to epinephrine, found that return of spontaneous circulation was achieved in six of eight episodes in patients treated with 15 to 20 mics/kg/dose of terlipressin and four of these patients survived without neurological sequelae.[47]

## RECOMMENDATION

In lieu of the above data, it can be summarised that vasopressin does have a role in cardiopulmonary resuscitation, particularly in asystole, and this needs to be evaluated further in the pediatric population, as vasopressin does have a superior role in maintaining vital organ perfusion as compared to epinephrine, in a recommended dosage.

## VASOPRESSIN DOSAGE AND SAFETY

To date, the most common use of vasopressin was as intravenous infusion for controlling gastrointestinal hemorrhage and for the treatment of diabetes insipidus.

The dose range is variable with the maximum dose up to 0.008 units/kg/min. The upper dose limit in adults with vasodilatory shock is 0.04 units/min. Doses beyond this range are not associated with increased effectiveness, although they are associated with increased adverse events.[48]

Reported adverse events related to exogenous vasopressin include coronary ischemia, increased myocardial afterload, ischemic skin lesions, wound complications, new onset tachy arrhythmias, and splanchnic hypoperfusion.[49] Complications are more common when there is coadministration of vasopressin and prolonged use of a moderate-to-high dose of norepinephrine.[50] The effect of vasopressin on coronary circulation remains an enigma, with contradictory studies.V1 receptor-mediated coronary vasoconstriction is a dose-dependent phenomenon that may be attenuated by endothelial vasodilatation, mediated via the oxytocin receptor or P2 receptor. In addition to its vascular effects on the coronary blood flow, vasopressin has mitogenic and metabolic effects on the heart.[51] On account of the potent vasoconstrictor action of vasopressin, the possibility of impaired capillary blood flow and tissue oxygenation with vasopressin administration, remains a concern. New techniques for assessing microcirculatory perfusion, such as orthogonal polarization spectral imaging, as in recent studies, provide invaluable information with respect to microcirculatory responses during vasopressor therapy.[52]

## CONCLUSION

Vasopressin is gaining popularity in diverse states such as septic shock and vasodilatory shock associated with cardiac anesthesia and surgery. We stress that the clinical studies to date have been small and have focused on physiological outcomes, and the data on adverse effects are limited. Therefore, we do not recommend vasopressin as a first-line therapy. Future prospective studies are necessary to define the role of vasopressin in different conditions. The use of vasopressin, like any empirical therapy, requires the assessment of therapeutic end points and surveillance of potential adverse effects, which are important tools during the titration of vasoactive therapy in critically ill patients.[53]
